#  Persistent negative visual aura in migraine without headache: a case report

**DOI:** 10.1186/1752-1947-8-61

**Published:** 2014-02-19

**Authors:** Jisun Lim, Kwang Deog Jo, Moon Kyu Lee, Wooyoung Jang

**Affiliations:** 1 Department of Family Medicine, Asan Medical Center, University of Ulsan College of Medicine, Seoul, Republic of Korea; 2 Department of Neurology, Gangneung Asan Hospital, University of Ulsan College of Medicine, Bangdong-ri, Sacheon-myeon, Gangneung-si, Gangwon-do 210-711, Republic of Korea

**Keywords:** Aura, Cortical spreading depression, Criteria, Headache, Migraine

## Abstract

**Introduction:**

Persistent migraine aura without headache is an extremely rare condition. The International Headache Society defines various subtypes of migraines, including “persistent migraine aura without infarction” and “typical aura without headache.”

**Case presentation:**

We describe the case of a 21-year-old Asian woman with a history of migraine without aura who had (as her first aura episode) persistent negative visual symptoms without headache for 6 months. We detected no lesions that could cause her persistent visual symptoms. Based on the patient’s history of migraine without aura and responsiveness to furosemide and lamotrigine, we concluded that the visual symptoms in this case were related to migraine visual auras.

**Conclusions:**

Persistent visual aura without headache overlapped the criteria for the two migraine subtypes mentioned above and thus did not fit an exact diagnosis. Therefore, we assert that new criteria are needed to encompass uncertain visual symptoms of migraine aura.

## Introduction

Migraine is a common aetiology of headache that causes functional disability [1]. A migraine aura can be defined as a neurological disturbance that emerges prior to or during the appearance of a migraine headache [1]. The second edition of the International Classification of Headache Disorders (ICHD-II) divided migraine into various subtypes [2]. Among these subtypes, migraine aura without headache is a relatively uncommon phenomenon, with a lifetime prevalence of 3% in women and 1% in men [3]. Some patients with migraine present with a continuous visual phenomenon, and no specific aetiology has been found for these patients despite detailed investigation. ICHD-II defines such cases as persistent migraine aura without infarction, which is a rare but well-documented condition [2]. These two subtypes, that is, migraine aura without headache and persistent migraine aura without infarction, are examples of the rare end of the migraine spectrum. To the best of our knowledge, few reports have addressed migraine symptoms that overlap these two subtypes.

Here we describe the case of a patient with a persistent negative visual aura without migraine headache.

## Case presentation

A 21-year-old Asian woman presented (via an ophthalmologist) with a 6-month history of the right side of her field of vision going dark. This visual impairment caused her to frequently fall out of bed or off chairs when rising from them. This first-episode visual symptom was constant and was not accompanied by any positive visual phenomena. She had no previous history of visual aura and experienced no tingling, numbness or weakness of the limbs. She had a history of headache from the age of 13, occurring at a frequency of once per month, as is consistent with typical migraines without auras. This symptom was relieved by acetaminophen, and she received no prophylactic medication for her migraines. She had no migraine headaches when her persistent visual symptom began. There was no history of tobacco, alcohol or illicit drugs. Her medical history was also unremarkable. No family history of migraine or other neurological diseases was identified.

**Figure 1:**
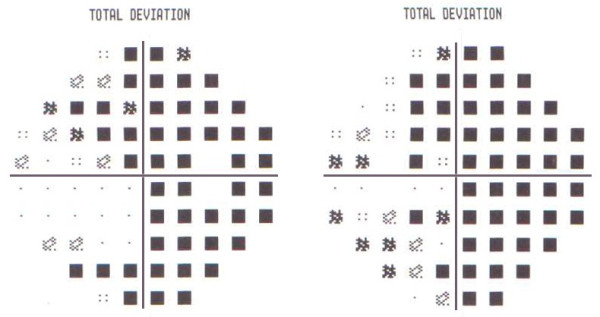
Humphrey 20–2 SITA-Fast Threshold visual field test revealed complete right homonymous hemianopsia.

Physical and ophthalmological examinations including visual acuity, colour discrimination and retinogram were normal. However, a hemifield defect was apparent in a confrontation test. This hemianopsia was confirmed in an ophthalmologic examination using computerised campimetry (Figure 1). Further neurological examination revealed no disturbance. All laboratory investigations including complete blood count and chemistry, iron studies and autoantibody evaluation were negative. Magnetic resonance imaging (MRI) and magnetic resonance angiography did not identify any potential cause for the visual disturbance. Electroencephalogram visual evoked potential, transcranial Doppler (TCD) for a patent foramen ovale test and a TCD base scan also showed normal results. Her brain perfusion single-photon emission computed tomography (SPECT) was unremarkable. She was prescribed lamotrigine at a dose of 25mg and furosemide at a dose of 40mg. Two weeks later, she reported a significant improvement after medication administration.

## Discussion

Auras associated with migraines are reported in approximately 20% of migraineurs, and nearly all auras present as visual symptoms [4]. Common descriptions of visual aura consist of various positive or negative phenomena including bright spots, flashes of light or scintillating scotoma [1,5]. Homonymous hemianopsia related to migraine has been well documented, and Goodwin recently described a complete transient homonymous hemianopsia in a migraine attack similar to our case [5].

Auras are persistent visual symptoms that can last several days up to a number of months. A persistent migraine aura without infarction is a specific diagnosis according to the following International Headache Society classification ICHD-II criteria: 1) previous attacks fulfilling the criteria for migraines with auras, 2) the present attack is similar to previous attacks but one or more aura symptoms persist for more than 2 weeks, and 3) the aura is not attributed to another disorder [2]. Although the most important point of diagnosis is the exclusion of structural or vascular lesions, the diagnosis of persistent migraine aura without infarction in this case presented some challenging problems.

First, the diagnostic criteria are somewhat strict. Our patient should have had a history of migraine with aura prior to the diagnosis of a persistent migraine aura without infarction. However, our patient reported a persistent migraine visual aura for 6 months and did not have a typical history of migraine with aura. Similar cases have been reported. Liu *et al*. described 10 patients who had persistent visual auras over long durations [6]. Among these patients, seven had a previous history of migraine with aura. However, three patients had migraine without aura. Almeida *et al*. also reported one case of migraine with persistent visual aura with a 7-year history of migraine without aura. This patient then twice experienced persistent visual symptoms [7]. Because the ICHD-II criteria require a history of migraine with aura, none of these cases fulfilled the diagnostic criteria for persistent migraine aura without infarction. Therefore, the ICHD-II criteria leave a grey area in the diagnosis of persistent migraine aura without infarction, and long-term follow-up is needed to confirm the diagnosis.

Second, our patient did not have migraine headaches during her persistent visual symptoms, which does not match the ICHD-II diagnostic criteria for persistent migraine aura without infarction: “the present attack is typical of previous attacks” (previous attacks fulfil the criteria for migraine with aura). Thus, we considered other criteria. ICHD-II describes other rare conditions, such as typical aura without headache which is defined as follows: two or more episodes of aura without headache, the aura is fully reversible, the aura is indicative of a focal cortical dysfunction, at least one aura evolves over 5 minutes, two or more symptoms occur in succession, the duration of the symptoms is less than 60 minutes and the patient’s history and examination are both negative for other aetiologies [2]. Our case also did not meet these criteria because she reported that the prolonged visual aura was her first episode.

Our patient had a typical history of migraine without aura, and no other aetiologies were revealed. In an ophthalmologic evaluation, a binocular hemifield defect was demonstrated. It therefore appeared that the visual symptom of our case was associated with a migraine aura. This case was an extremely rare example of an isolated persistent visual aura that could not be classified into a specific migraine subtype. Thus, we suggest that ICHD-II criteria should be altered to encompass this uncertain visual symptom related to migraine aura.

The cause of visual aura in migraine has long been of interest, and vascular and neuronal theories have been proposed [8]. In the vascular theory (proposed by Wolff), the neurologic symptoms of the migraine aura are caused by cerebral vasoconstriction [4]. Another theory is based on cortical spreading depression (CSD), which is a neuroelectric event beginning in the occipital cortex that propagates into contiguous brain regions. Using functional MRI techniques, Hadjikhani *et al*. found that some patients with aura experienced a slow neuronal change in the occipital cortex that moved forward at a rate of 3 to 6mm per minute [9]. Chen *et al*. recently reported that the visual cortex in patients with persistent visual aura maintained a steady-state hyperexcitability, which suggests reverberating CSD [10]. However, our case demonstrated no SPECT abnormality, which indicates that other mechanisms of visual aura could be involved in persistent visual aura with migraine.

Empirical medications have been used to treat persistent visual aura in migraine including cyproheptadine, dihydroergotamine, furosemide, lamotrigine and nimodipine [7]. The mechanisms underlying the therapeutic effects are based on the neuronal theory of CSD. We used furosemide and lamotrigine to treat our patient. Furosemide affects the levels of potassium, which is necessary for the initiation of CSD; lamotrigine also acts on CSD. The improvement of visual aura after the administration of these medications provides further evidence that the persistent visual symptoms in our patient may be related to migraine aura.

## Conclusions

Our patient demonstrated an extremely rare spectrum of migraine aura. Although ICHD-II criteria can be used to define a wide spectrum of migraines, our case did not fit any specific subtype used by ICHD-II. Thus, we suggest that a specific migraine subtype encompassing persistent visual aura without headache should be included in the ICHD-II criteria.

## Consent

Written informed consent was obtained from the patient for publication of this case report and accompanying images. A copy of the written consent is available for review by the Editor-in-Chief of this journal.

## Abbreviations

CSD: Cortical spreading depression; ICHD-II: International classification of headache disorders; MRI: Magnetic resonance imaging; SPECT: Single-photon emission computed tomography; TCD: Transcranial doppler.

## Competing interests

The authors declare that they have no competing interests.

## Authors’ contributions

JL carried out the research project (conception, organisation and execution), statistical analysis (design and execution) and wrote the first draft. KDJ carried out the research project (execution and acquisition of data) and reviewed and critiqued this report. MKL carried out the research project (execution and acquisition of data) and reviewed and critiqued this report. WJ carried out the research project (conception, organisation, and execution), statistical analysis (design, execution and review), drafted, reviewed and critiqued this report. All authors read and approved the final manuscript.
